# Modelling tuberculosis transmission dynamics in Malaysia

**DOI:** 10.3389/fpubh.2026.1843471

**Published:** 2026-07-14

**Authors:** Mei Cheng Lim, Nur’ain Mohd Ghazali, Mohd Kamarulariffin Kamarudin, Nuur Hafizah Md Iderus, Sumarni Mohd Ghazali, Mohd Azahadi Omar, Suzana Mohd Hashim, Siti Hafsah Abdul Halim, Shazelin Alipitchay, Asmah Razali, Siti Roszilawati Ramli, Balvinder Singh Gill, Norazaliza Mohd Jamil, Sarat Chandra Dass, Sarbhan Singh

**Affiliations:** 1 Special Resource Centre, Institute for Medical Research, National Institutes of Health, Ministry of Health, Shah Alam, Malaysia; 2 Tuberculosis and Leprosy Control Sector, Disease Control Division, Ministry of Health, Putrajaya, Malaysia; 3 Infectious Disease Research Centre, Institute for Medical Research, National Institutes of Health, Ministry of Health, Shah Alam, Malaysia; 4 National Public Health Laboratory, Ministry of Health, Sungai Buloh, Malaysia; 5 Centre for Mathematical Sciences, Universiti Malaysia Pahang Al-Sultan Abdullah, Kuantan, Malaysia; 6 School of Mathematical and Computer Sciences, Heriot-Watt University Malaysia, Putrajaya, Malaysia

**Keywords:** compartmental model, end TB strategy, Malaysia, reproduction number, transmission dynamics, tuberculosis

## Abstract

**Introduction:**

Tuberculosis (TB) remains a major global public health challenge requiring urgent interventions to achieve the World Health Organization (WHO) End TB Strategy targets. This study aimed to develop and validate a compartmental model to estimate the TB reproduction number (R_0_) and TB incidence in Malaysia, identify parameters influencing R_0_ and TB incidence, and simulate TB incidence using baseline and intervention scenarios to 2035 to assess compatibility with achieving the WHO End TB Strategy target.

**Methods:**

A Susceptible-Latent Fast-Latent Slow-Infectious-Recovered (SL_F_L_S_IR) compartmental model was developed and calibrated using national TB data from 2013 to 2023. Model calibration was performed using initial Monte Carlo exploration followed by Particle Swarm Optimisation to identify final calibrated parameters. R_0_ was estimated using the next-generation matrix approach. Uncertainty was quantified using parametric bootstrapping with negative binomial observation model. Model validation used observed TB cases in 2024 and 2025 and was assessed using performance metrics. Global sensitivity analysis using Latin Hypercube Sampling and Partial Rank Correlation Coefficients identified parameters influencing R_0_ and simulated TB incidence in 2035. TB incidence scenarios were simulated to 2035 by varying the transmission rate (*β*), rate of progression from active TB to recovery (*γ*), and rate of progression from latent fast to active TB (*ε*).

**Results:**

The calibrated model-estimated TB cases and incidence showed good fit to observed TB cases and incidence during calibration and short-term validation periods, with satisfactory performance metrics. The estimated R_0_ was 1.09 (95% CI: 1.01–1.27). Parameters influencing R_0_ were *β* and *γ*, while parameters influencing simulated TB incidence in 2035 were *β*, *γ*, and rate of progression from latent slow to active TB (*κ*). TB incidence showed minimal reduction by 2035 in the baseline scenario. The most intensive intervention scenario reduced TB incidence by 66.21% by 2035, relative to 2015.

**Conclusion:**

The SL_F_L_S_IR model showed that TB transmission in Malaysia remains self-sustaining, with estimated R_0_ slightly above 1. Although combined TB-specific interventions could reduce simulated TB incidence, achieving the WHO End TB Strategy targets by 2035 will likely require integrated TB control with broader improvements in population health and social determinants of TB risk.

## 1 Introduction

Tuberculosis (TB) remains one of the oldest yet enduring challenges for global public health despite decades of progress in TB control and management. According to the 2025 WHO Global Tuberculosis Report, the global TB incidence in 2024 was estimated at 10.7 million cases, equivalent to 131 new cases per 100,000 population (1). The WHO End TB Strategy sets the goal of a 90% reduction of TB incidence by 2035 (compared to 2015). However, the net global decline in TB incidence between 2015 and 2024 was only 12%, which is substantially below the interim milestone of a 50% reduction by 2025 (1). While 30 countries, mostly in African and European regions, have met the 2025 End TB milestone of more than 50% reduction in incidence, the majority of countries worldwide have yet to attain this milestone, including Malaysia (2). Nevertheless, there remains a window of opportunity until 2035 to reach the targets.

Attaining these goals will require countries to adhere to several core principles of the End TB Strategy: (i) government stewardship and accountability with robust monitoring and evaluation; (ii) strong engagement of civil society organisations and communities; (iii) protection and promotion of human rights, ethics, and equity; (iv) adaptation of the strategy at national level with global collaboration (1, 3). The success of these strategies relies on many factors, among them is a clear understanding of local TB transmission dynamics, specifically the reproduction number (R_0_).

R_0_ is one of the fundamental metrics in infectious disease epidemiology, defined as the average number of secondary infections generated by a single infectious case in a fully susceptible population (4). It provides essential information on the disease’s transmission potential and the likelihood of sustained spread. When R_0_ is greater than 1, transmission is expected to continue, and the disease may expand within the population, whereas values below 1 indicate that transmission will gradually decline and the disease will eventually die out (4–6). Estimating R_0_ therefore provides a quantitative benchmark for underlying transmission potential of TB in a population.

Previous studies conducted in China, India, Indonesia, Taiwan, the Netherlands, the United States, South Africa, Ukraine and Malaysia have reported TB R_0_ estimates ranging from 0.24–4.3 (7–9). The variation in TB R_0_ estimates is attributed to several factors, including the methods used for estimating R_0_. Given the context-specific nature of R_0,_ a range of methodological approaches have been employed to estimate TB transmission dynamics. Empirical methods based on contact-tracing data infer transmission by counting secondary cases arising from identified contacts (10, 11). For example, Borgdorff et al. combined TB bacterial DNA fingerprinting with surveillance data to identify epidemiologically linked cases and infer recent transmission to derive R_0_ (12). While informative, such approaches require long-term follow-up, high-quality laboratory infrastructure and comprehensive surveillance systems, which are often unavailable or incomplete in many settings. In addition, empirical R_0_ measurement for TB is challenging due to its long and variable latency period and the difficulty of directly observing transmission events (4, 5). Therefore, R_0_ is commonly estimated through mathematical models (4, 5).

Mathematical models such as compartmental models provide a systematic and flexible framework for estimating R_0_ by translating the biological and epidemiological processes of disease transmission into a system of mathematical equations. In these models, the population is stratified into compartments representing disease states, such as susceptible, latent, infectious, and recovered, with transitions between states governed by differential equations. From these equations, the R_0_ can be derived analytically to quantify the expected number of new infections from a single infectious individual in the population of interest (6, 9, 13). Numerous studies have successfully applied compartmental models to estimate TB transmission dynamics across diverse settings (9, 14–16).

Beyond estimating R_0_, compartmental models can also be used to assess how potential interventions may influence TB incidence. In TB modelling, changes in transmission, progression from latent infection to active disease, and recovery from active TB can be linked to major intervention domains, including active case detection, earlier diagnosis and treatment, improved treatment completion, transmission reduction, and LTBI screening and preventive treatment (9, 17–19). Scenario simulations are therefore useful for assessing whether plausible intervention combinations could shift incidence trajectories towards the WHO End TB Strategy target (9, 17, 18).

TB remains an important public health concern in Malaysia, and several compartmental modelling studies have examined TB transmission in the Malaysian context. Md Nor et al. applied Susceptible-Infected-Recovered model with and without demographic factors, using the Runge–Kutta method to predict TB transmission in Malaysia and simulated changes under different transmission and recovery rates (20). Tamhaji and Hamdan developed a BCG vaccinated-Susceptible-Exposed-Infected-Recovered model incorporating immigration, and reported R_0_ estimates under different immigration assumptions (8). More recently, Hamdan et al. used a Susceptible-Vaccinated-Infectious-Recovered framework to examine the role of vaccination in TB transmission in Malaysia (21). Collectively, these studies illustrated the use of compartmental models to examine TB transmission dynamics in Malaysia.

Building on this existing work, the present study developed and calibrated a model using national TB data, incorporating fast and slow latent TB progression within a population-level modelling framework. In this context, this study aimed (i) to develop and validate a compartmental model to estimate TB R_0_ and TB incidence in Malaysia; (ii) to identify parameters influencing TB R_0_ and TB incidence; (iii) to simulate TB incidence using baseline and intervention scenarios until 2035 to assess whether simulated trajectories are compatible with achieving the WHO End TB Strategy target.

## 2 Materials and methods

### 2.1 Data sources

In Malaysia, all confirmed TB cases must be notified to the District Health Office within 7 days of diagnosis under the Prevention and Control of Infectious Diseases Act, 1988 (Act 342) (22). Confirmed TB cases include bacteriologically confirmed, clinically diagnosed, pulmonary, extrapulmonary and relapse TB, which are defined according to the Case Definitions for Infectious Diseases in Malaysia, 2017 (23). For the study, annual data on the number of confirmed TB cases, TB deaths and recovered cases from 2013 to 2023 were sourced from the TB surveillance database maintained by the Disease Control Division, Ministry of Health (MOH), Malaysia. Local data of mid-year population estimates, crude birth rates and crude death rates from 2013 to 2023 were obtained from the Department of Statistics Malaysia (DOSM) (24).

### 2.2 Model formulation

The compartmental model was developed in R programming software using the *deSolve* package, which provides numerical solvers for systems of differential equations (25). The model structure was adapted from the classic Susceptible-Infectious-Recovered (SIR) framework originally formulated by Kermack and McKendrick in 1927, with extensions to reflect key features of TB natural transmission dynamics, resulting in the Susceptible-Latent Fast-Latent Slow-Infectious-Recovered (SL_F_L_S_IR) model (13, 26).

**Figure 1:**
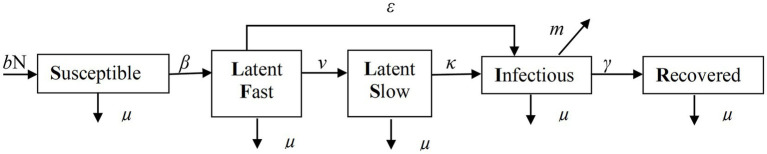
SL_F_L_S_IR compartmental model.

The SL_F_L_S_IR model comprises five compartments: Susceptible (*S*), Latent Fast (*L_F_*), Latent Slow (*L_S_*), Infectious (*I*), and Recovered (*R*), as illustrated in Figure 1. The *S* compartment represents uninfected individuals fully susceptible to TB infection. Upon exposure to an infectious individual, the individuals in *S* can become infected with latent TB infection (LTBI) and move to the *L_F_* compartment at a rate governed by the transmission parameter (*β*). The *L_F_* compartment represents individuals with recent LTBI during the first 2 years after infection, a period when progression risk to active TB is highest. In this model, the transition from *L_F_* to *L_S_* was governed by the rate *ν* to represent an average two-year transition from recent to longer-term latent infection. The *L_S_* compartment represents longer-term LTBI beyond the recent latent phase, with lower but persistent risk of reactivation. This structure was used to distinguish rapid progression following recent infection from slower progression to active TB during longer-term latent infection (27, 28). Progression to active TB into the *I* compartment occurs from *L_F_* at rate *ε* and from *L_S_* at rate *κ*, while those who do not progress to active TB remain in latent phase for prolonged periods. The *I* compartment represents individuals with active TB who are capable of transmitting infection. From this state, individuals either succumb to TB at a disease-specific mortality rate *m* or recover at rate *γ* and move into the *R* compartment.

To reflect the population dynamics throughout the modelling period, the model incorporates annual birth rates (*b*) and death rates (*μ*), with *μ* applied uniformly across all compartments as all individuals are subjected to the same background death rate. The system of ordinary differential equations described below was solved numerically using *deSolve* (Equation 1).


$$\frac{\mathrm{dS}}{\mathrm{dt}}=\mathrm{bN}-\beta \frac{S}{N}I-\mathrm{\mu S}$$

$$\frac{dL_{F}}{\mathrm{dt}}=\beta \frac{S}{N}I-\varepsilon L_{F}-\nu L_{F}-\mu L_{F}$$

$$\frac{dL_{S}}{\mathrm{dt}}=\nu L_{F}-\kappa L_{S}-\mu L_{S}$$ (1)

$$\frac{\mathrm{dI}}{\mathrm{dt}}=\varepsilon L_{F}+\kappa L_{S}-\mathrm{\gamma I}-\mathrm{mI}-\mathrm{\mu I}$$

$$\frac{\mathrm{dR}}{\mathrm{dt}}=\mathrm{\gamma I}-\mathrm{\mu R}$$

The SL_F_L_S_IR model had the following assumptions: (i) The population mixes homogeneously, and all individuals have an equal likelihood of contracting and transmitting the infection during the active infectious period (29, 30). (ii) Individuals with LTBI in *L_F_* and *L_S_* are not infectious. (iii) Recovered individuals are not susceptible to reinfection of TB within the modelled period (29, 30).

### 2.3 Model calibration

The SL_F_L_S_IR model was calibrated to annual TB cases from 2013 to 2023. Before model calibration, the annual TB case counts for 2020 to 2022 were adjusted upward by 15% to account for the impact of the COVID-19 pandemic (31). This was done to approximate the underlying burden of TB during these periods.

Model calibration was conducted in two stages. The calibration focused on four transition parameters *β*, *ε*, *κ*, *γ*, and three estimated initial values of the latent fast compartment, *L_F_(0)*, latent slow compartment, *L_S_(0)* and infectious compartment, *I(0)*. For brevity, these four transition parameters and three estimated initial compartment values are hereafter referred to collectively as the seven calibrated model parameters. Fixed parameters used in the SL_F_L_S_IR model are presented in Table 1.

**Table 1:** SL_F_L_S_IR compartmental model fixed parameters.

Parameter	Definition	Values	Source
N	Population of Malaysia, 2013	30,200,000	(24)
*b*	Average annual crude birth rate, 2013–2023	0.01541	(24)
*μ*	Average annual crude death rate, 2013–2023	0.0054	(24)
*m*	Average annual TB disease-specific death rate, 2013–2023	0.1127	MOH
ν	Rate of progression from latent fast to latent slow	0.5	(27, 28)

In the first stage, broad initial parameter exploration was performed using Monte Carlo sampling to generate 50,000 parameter sets. Model parameters and initial compartment values were randomly sampled from independent uniform distributions within prespecified biologically plausible bounds informed by published literature and local epidemiological data to ensure that the search was restricted to epidemiologically feasible parameter space (32, 33). For each sampled parameter set, the SL_F_L_S_IR model was solved numerically using the *deSolve* package in R. The model-estimated TB case counts were iteratively compared against the observed case counts. The model output used for calibration was the model-estimated new active TB cases, defined as the inflow into the *I* compartment from the *L_F_* and *L_S_* compartments, $\varepsilon L_{F}+\kappa L_{S}$ . The calibration sought to minimise the sum of squared differences between the observed and model-estimated case counts using a least-squares objective function defined as (Equation 2):


$$L(\theta )=\sum_{j=1}^{11}{\left(I_{\mathrm{obs}}\;(t_{j})-I_{\mathrm{mod}}\;(t_{j})\right)}^{2}$$ (2)

Where *θ* is a vector of parameters estimated through calibration (*β, ε, κ, γ, L_F_(0), L_S_(0) and I(0)*), *I_obs_ (t_j_)* denotes observed annual TB cases in year t_j_, and *I_mod_ (t_j_)* represents model-estimated new TB cases in year t_j_, generated from the inflow into the *I* compartment through progression from the *L_F_* compartment ( $\varepsilon L_{F}$ ) and *L_S_* compartment ( $\kappa L_{S}$ ) using parameter set *θ*, with j representing the annual observation time points in the study period. The parameter sampling ranges used in Monte Carlo exploration and the selected candidate parameter values were included in Supplementary Table S1.

In the second stage, the parameter set with the lowest value of *L(θ)* from Monte Carlo sampling was subsequently used as an initial candidate for refinement using Particle Swarm Optimisation (PSO). PSO is a population-based global optimisation algorithm that iteratively searches for multiple candidate parameter combinations (particles) simultaneously and updates each particle’s position at every iteration based on its previous and current best performance within the swarm to identify the parameter set that minimises the least-squares objective function measuring the discrepancy between model outputs and observed data (34–36). PSO was implemented in R using the *psoptim* function. For each PSO run, the swarm size was set to 100 particles, and the maximum number of iterations was set to 1,000. PSO was repeated for 20 independent runs using different random seeds. The final calibrated parameter set was selected from the run that produced the lowest objective function value across all runs.

### 2.4 Estimation of TB incidence from calibrated model

The calibrated SL_F_L_S_IR model was subsequently used to estimate annual TB cases and population size from 2013 to 2023. Annual TB incidence was calculated by dividing the estimated TB cases each year by the corresponding estimated population, expressed per 100,000 population.

### 2.5 Estimation of reproduction number

The R_0_ for the SL_F_L_S_IR model was derived analytically using the next-generation matrix approach (6, 9, 13, 35). In this derivation, the infected-state vector was defined as ${\left(L_{F},L_{S},I\right)}^{T}$ , where $L_{F}$ and $L_{S}$ represent latent TB infection compartments and I represents active TB. Although individuals in $L_{F}$ and $L_{S}$ are not infectious, these compartments were included in the infected-state subsystem because they represent infected individuals who may subsequently progress to active TB. At the disease-free equilibrium, $L_{F}^{*}=L_{S}^{*}=I^{*}=0$ and $S^{*}/N=1$ . New infections were assumed to enter the $L_{F}$ compartment through transmission from individuals in the I compartment. The full derivation is provided in Supplementary Method S1. In the context of this study, the R_0_ represents the expected number of secondary infections produced by a single infectious individual introduced into a fully susceptible population under the assumptions of the calibrated SL_F_L_S_IR model during the period of infectiousness (37). It is determined by the rate of transmission, the probabilities of progression from latent compartments into active TB, and the duration of infectiousness (37). The formula for R_0_ (Equation 3) is as follows:


$$R_{0}=\frac{\beta }{\gamma +\mu +m}\cdot [\frac{\varepsilon }{\varepsilon +\nu +\mu }+\frac{\nu }{\varepsilon +\nu +\mu }\cdot \frac{\kappa }{\kappa +\mu }]\;$$ (3)

where the first term $\frac{\beta }{\gamma +\mu +m}$ represents the number of infections generated per infectious case during the infectious period, and the second bracketed term $\frac{\varepsilon }{\varepsilon +\nu +\mu }+\frac{\nu }{\varepsilon +\nu +\mu }\cdot \frac{\kappa }{\kappa +\mu }$ represents the probability that newly infected individuals become infectious through fast or slow latent pathways.

### 2.6 Uncertainty quantification

Uncertainty quantification was performed using a parametric bootstrap approach (38, 39). The calibrated model was used to generate expected annual TB cases, with overdispersion in the observed count data accounted for using a negative binomial observation model. A total of 2,000 bootstrap datasets were generated from the fitted negative binomial distribution. For each bootstrap dataset, the model was refitted by re-estimating the seven calibrated parameters. Percentile-based 95% confidence intervals (CIs) were derived for the calibrated parameters and R_0._ CIs for fitted TB case trajectories were obtained from the distribution of mean bootstrap model outputs, and the prediction intervals (PIs) were generated by simulating negative binomial predictive case counts from the refitted trajectories. The corresponding incidence CIs and PIs were obtained by converting the case trajectories to incidence per 100,000 population.

### 2.7 Model validation

Following parameter estimation, the calibrated SL_F_L_S_IR model was used to generate model-estimated TB cases for 2024 and 2025, which were validated against observed TB cases in the same period.

Several metrics were used to evaluate model performance during the calibration and validation periods. Mean error (ME) was used to assess the average direction of prediction error, indicating whether the model tended to overestimate or underestimate observed TB cases. Mean absolute error (MAE) was used to quantify the average absolute difference between observed and model-estimated TB cases. Root mean squared error (RMSE) was used to give greater weight to larger deviations between observed and model-estimated values. Mean absolute percentage error (MAPE) was used to express prediction error as a percentage of observed cases, allowing interpretation of model performance relative to the size of the annual TB cases.

In addition, prediction interval (PI) coverage was assessed by checking whether the observed TB cases in 2024 and 2025 fell within the model’s 95% PI. This provided an indication of whether the observed values were consistent with the range of uncertainty generated by the model during the validation period.

### 2.8 Sensitivity analysis

To investigate the relative influence of model parameters on key epidemiological outputs, global sensitivity analysis was performed using Latin Hypercube Sampling (LHS) and Partial Rank Correlation Coefficients (PRCC) (32, 40). The analysis aimed to identify the parameters influencing R_0_ and TB incidence in 2035. Although annual TB cases were also generated by the model, TB incidence was selected as the main output as it accounts for population size and corresponds directly to the WHO End TB Strategy incidence reduction target.

LHS was used to generate 2,000 parameter combinations by varying seven calibrated model parameters, *β, γ, ε, κ, L_F_(0), L_S_(0)* and *I(0),* across predefined plausible ranges around the calibrated parameter estimates. This approach allows for efficient exploration of the multidimensional parameter space by stratifying each parameter range into intervals of equal probability and sampling once from each interval. For each sampled parameter set, the SL_F_L_S_IR model was simulated from 2013 to 2035.

PRCC was used to assess monotonic associations between sampled parameters and model outputs, while adjusting for the influence of other sampled parameters. PRCC was calculated by rank-transforming the sampled parameters and model outputs, regressing each ranked parameter and ranked output on the remaining ranked parameters, and estimating the Pearson correlation between the resulting residuals. PRCC values range from −1 to +1, with positive and negative values indicating increasing and decreasing effects on output, respectively, while larger absolute PRCC values indicate stronger relative influence on the model output.

### 2.9 Scenario simulations

Following model calibration and validation, the SL_F_L_S_IR model developed was used to simulate the potential effects of TB interventions on TB incidence in Malaysia. Scenario simulations were conducted to assess whether different intervention combinations could achieve the WHO End TB Strategy targets of a 90% reduction in TB incidence by 2035 relative to 2015 levels or reduce TB incidence to below 10 cases per 100,000 population. Scenario simulations focused on changes in three parameters, namely transmission rate (*β*), rate of progression from latent fast to active TB (*ε*) and rate of progression from active TB to recovery (*γ*). These parameters were selected as they directly correspond to major TB control strategies outlined in the National Strategic Plan to End TB (2021–2030), namely transmission reduction, improvement of the TB care cascade, and LTBI management (41).

Reductions in *β* were used to represent interventions that reduce community transmission, including intensified case detection through active screening and case finding, expanded radiological and laboratory diagnostic capacities, and earlier diagnosis of infectious TB cases. Increases in *γ* were used to represent interventions that shorten the duration of infectiousness through earlier treatment initiation, improved treatment adherence and completion, and enhanced access to TB care services. Because these strategies are closely linked along the TB care cascade, *β* and *γ* were varied jointly in the scenario simulations. In this context, improvement in *β* and *γ* refers to a reduction in the transmission rate (*β*) and an increase in the recovery rate (*γ*), respectively.

In contrast, reductions in *ε* were used to represent LTBI-focused interventions that reduce progression from recent latent infection to active TB. These include expanded LTBI screening, improved LTBI diagnosis, and provision of TB preventive treatment among eligible high-risk groups. Therefore, *ε* was varied independently to represent different intensities of LTBI-focused intervention.

The rate of progression from *L_S_* to active TB (*κ*) was not varied in the scenario simulations. This parameter represents longer-term progression or reactivation from latent slow infection to active TB, which is influenced by broader host and population-level determinants and less directly modifiable through TB programme activities alone.

Based on this framework, scenario simulations were structured as follows (Table 2):

- Scenario 1 (Baseline): No changes to *ε*, *β* and *γ.*
- Scenario 2(a)–2(c) (Intervention scenarios): Fixed reductions in *ε* at 25, 50 and 75%, with corresponding simultaneous improvements in *β* and *γ* at 0, 25, 50 and 75% within each *ε* scenario.

**Table 2:** Baseline and intervention scenarios simulation.

Scenario	Reduction in *ε* (%)	Improvements in *β* and *γ* (%)
Scenario 1 (Baseline)	0	0
Scenario 2 (Intervention)		
2(a)	25	0, 25, 50, 75
2(b)	50	0, 25, 50, 75
2(c)	75	0, 25, 50, 75

ε is the rate of progression from latent fast to active TB; β is transmission rate; γ is the rate of progression from active TB to recovery.

The simulation period was from 2026 to 2035, during which the intervention effects were assumed to scale up gradually from 2026, reach target levels by the end of 2028, and remain constant until 2035. These scenarios were intended as exploratory counterfactual simulations to assess the level of intervention intensity required to substantially reduce TB incidence.

## 3 Results

### 3.1 Model calibration using TB cases

**Figure 2:**
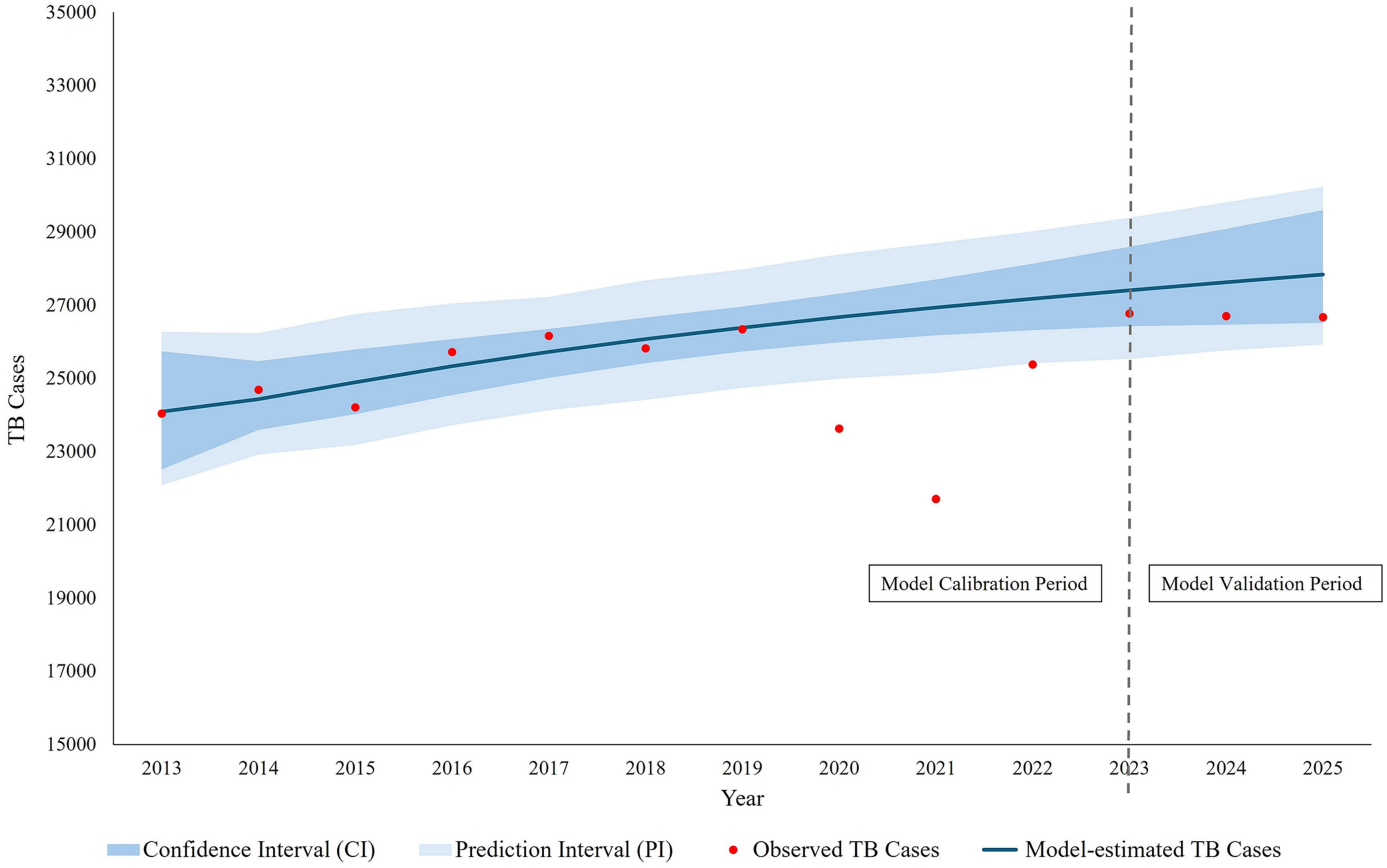
Calibration and validation of SL_F_L_S_IR model, 2013–2025.

During the calibration period, the model-estimated TB cases followed the overall upward trend of observed TB cases, as shown in Figure 2. The observed TB cases were generally well aligned with the model-estimated values and remained within the 95% PI, except for 2020 and 2021.

The calibration performance metrics demonstrated ME of 0.37, suggesting minimal average directional bias across the calibration period. The MAE was 658 cases, indicating an average absolute difference of approximately 658 cases per year. The RMSE was 931 cases, suggesting the presence of larger deviations in some years, particularly during 2020 and 2021. The MAPE was 2.49%, indicating a small relative difference between observed and model-estimated TB cases during the calibration period.

Overall, these findings suggest that the calibrated SL_F_L_S_IR model reproduced the observed TB case trend reasonably well during the calibration period. The calibrated parameter values of the SL_F_L_S_IR model are shown in Table 3.

**Table 3:** SL_F_L_S_IR compartmental model calibrated parameters.

Parameter	Definition	Values (95% CI)
*β*	Transmission rate	0.58 (0.36–1.00)
*ε*	Rate of progression from latent fast to active TB	0.51 (0.25–0.97)
*κ*	Rate of progression from latent slow to active TB	0.06 (0.03–0.10)
*γ*	Rate of progression from active TB to recovery	0.39 (0.20–0.72)
*L_F_(0)*	Initial value of latent fast compartment	23,146 (16,592-29,710)
*L_S_(0)*	Initial value of latent slow compartment	216,294 (154,007-277,640)
*I(0)*	Initial value of infectious TB compartment	41,631 (29,817-53,576)

The calibrated parameters should be interpreted as model-dependent estimates and not direct epidemiological estimates.

### 3.2 Estimation of TB incidence and R_0_

**Figure 3:**
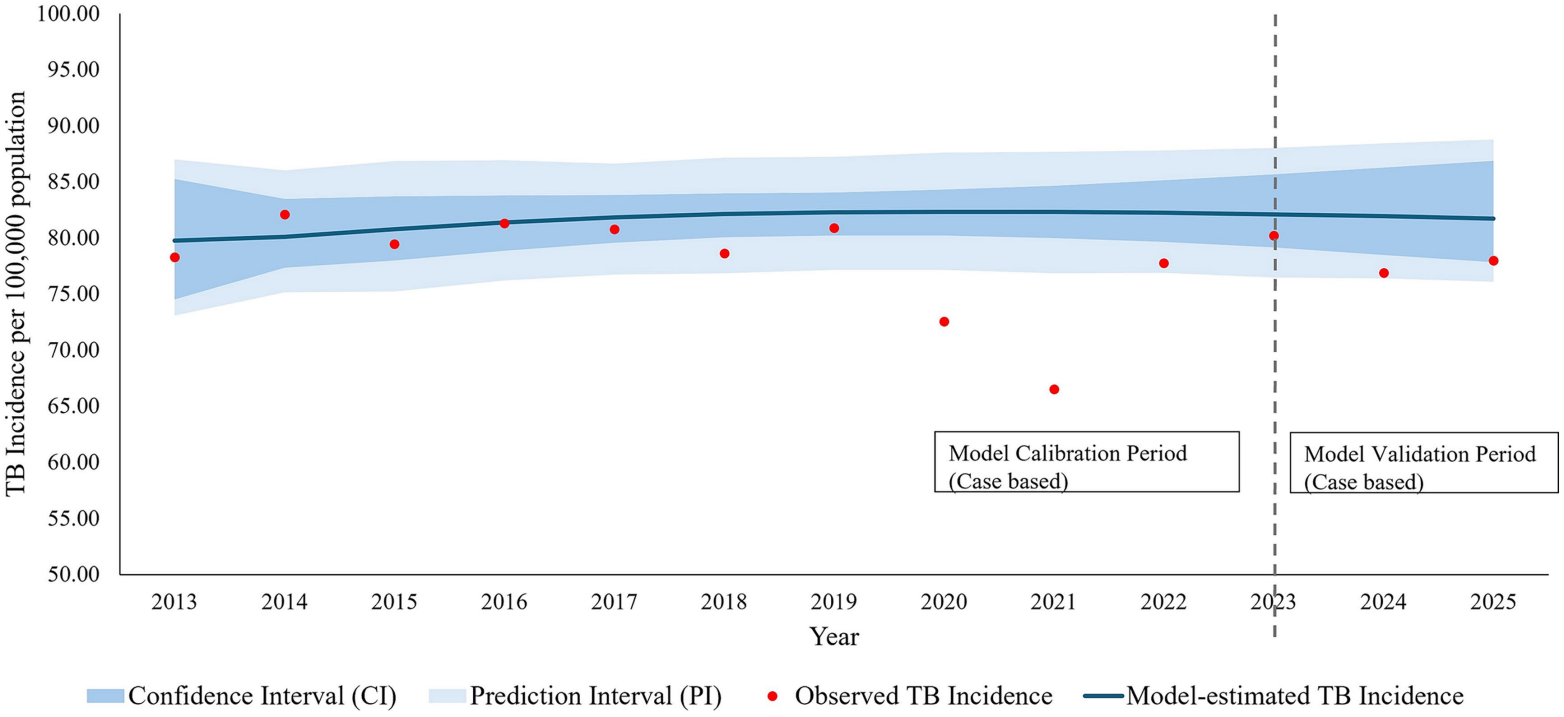
SL_F_L_S_IR model-estimated TB incidence per 100,000 population, 2013–2025.

The model estimated TB incidence per 100,000 population closely mirrored observed TB incidence from 2013 to 2025 (Figure 3). Overall, the estimated TB incidence trajectory showed a largely stable TB burden over the study period. Observed TB incidence values were in close alignment with the model estimates, as they were well within the 95% PI, except for 2020 and 2021. Based on the calibrated SL_F_L_S_IR model, the R_0_ for TB in Malaysia was estimated to be 1.09 (95% CI: 1.01–1.27).

### 3.3 Model validation

During the model validation period, the model estimated 27,624 TB cases in 2024 and 27,833 TB cases in 2025, compared with observed TB cases of 26,703 and 26,672, respectively. This corresponds to absolute differences of 921 cases in 2024 and 1,161 cases in 2025, equivalent to percentage differences of 3.45 and 4.35%, respectively (Figure 2).

The model-estimated TB cases were slightly higher than the observed values in both validation years, indicating a modest tendency towards overestimation during the short-term validation period. This was reflected in the ME of 1,041 cases. The MAE was also 1,041 cases, indicating an average absolute difference of approximately 1,000 cases across the two validation years. The RMSE was 1,047 cases, close to the MAE, suggesting broadly similar error magnitude in 2024 and 2025. The MAPE was 3.90%, suggesting a small relative difference between model-estimated and observed TB cases during the validation period.

Although the model estimates were slightly higher than the observed TB cases in 2024 and 2025, the observed values remained within the model’s 95% prediction interval. This suggests that the calibrated model trajectory remained broadly consistent with observed TB cases outside the calibration period. Overall, the model demonstrated reasonable short-term consistency with observed TB data, although validation was limited to two annual observations.

### 3.4 Sensitivity analysis

**Figure 4:**
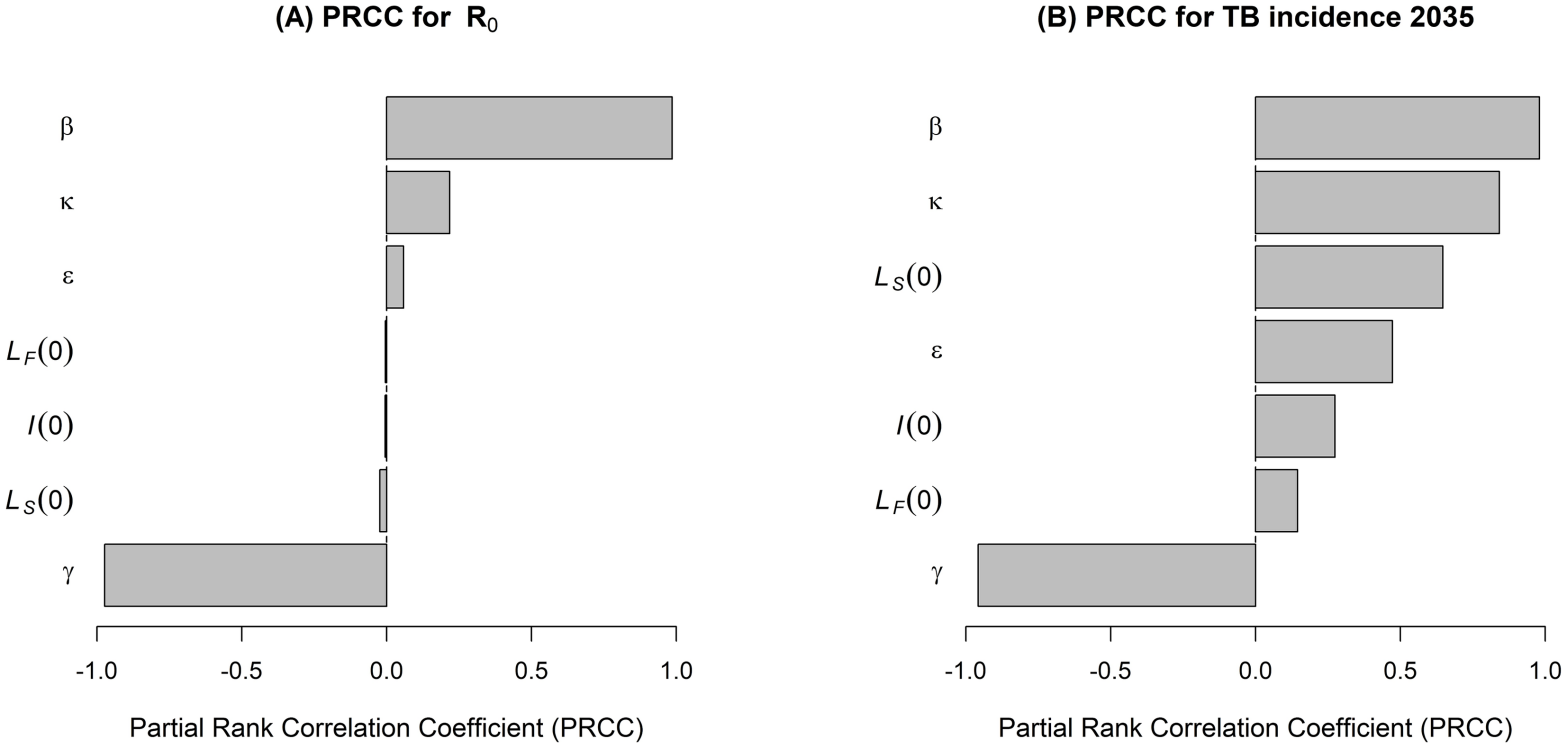
Global sensitivity analysis using LHS-PRCC for **(A)** R_0_ and **(B)** TB incidence in 2035.

In the LHS PRCC sensitivity analysis, 2,000 sampled parameter sets were generated to assess the influence of calibrated model parameters on R_0_ and TB incidence in 2035. For R_0_, *β* showed the strongest positive association (PRCC = 0.98), *γ* showed the strongest negative association (PRCC = −0.97) and *κ* showed a weak positive association with R_0_ (PRCC = 0.22; Figure 4). These findings indicate that higher transmission increased R_0_ whereas faster recovery or removal from infectiousness reduced transmission potential.

For TB incidence in 2035, *β* showed the strongest positive association (PRCC = 0.98), while *γ* showed the strongest negative association (PRCC = −0.96). In addition, *κ* also showed a strong positive association (PRCC = 0.84), followed by *L_S_(0)* (PRCC = 0.65), and *ε* (PRCC = 0.47). However, *I(0)* showed a weak association (PRCC = 0.27), while *L_F_(0)* showed negligible association (PRCC = 0.14; Figure 4). These findings suggest that TB incidence was influenced by both ongoing transmission and latent infection dynamics.

### 3.5 Scenario simulation of TB incidence (2026–2035)

#### 3.5.1 Scenario 1 (baseline)

**Figure 5:**
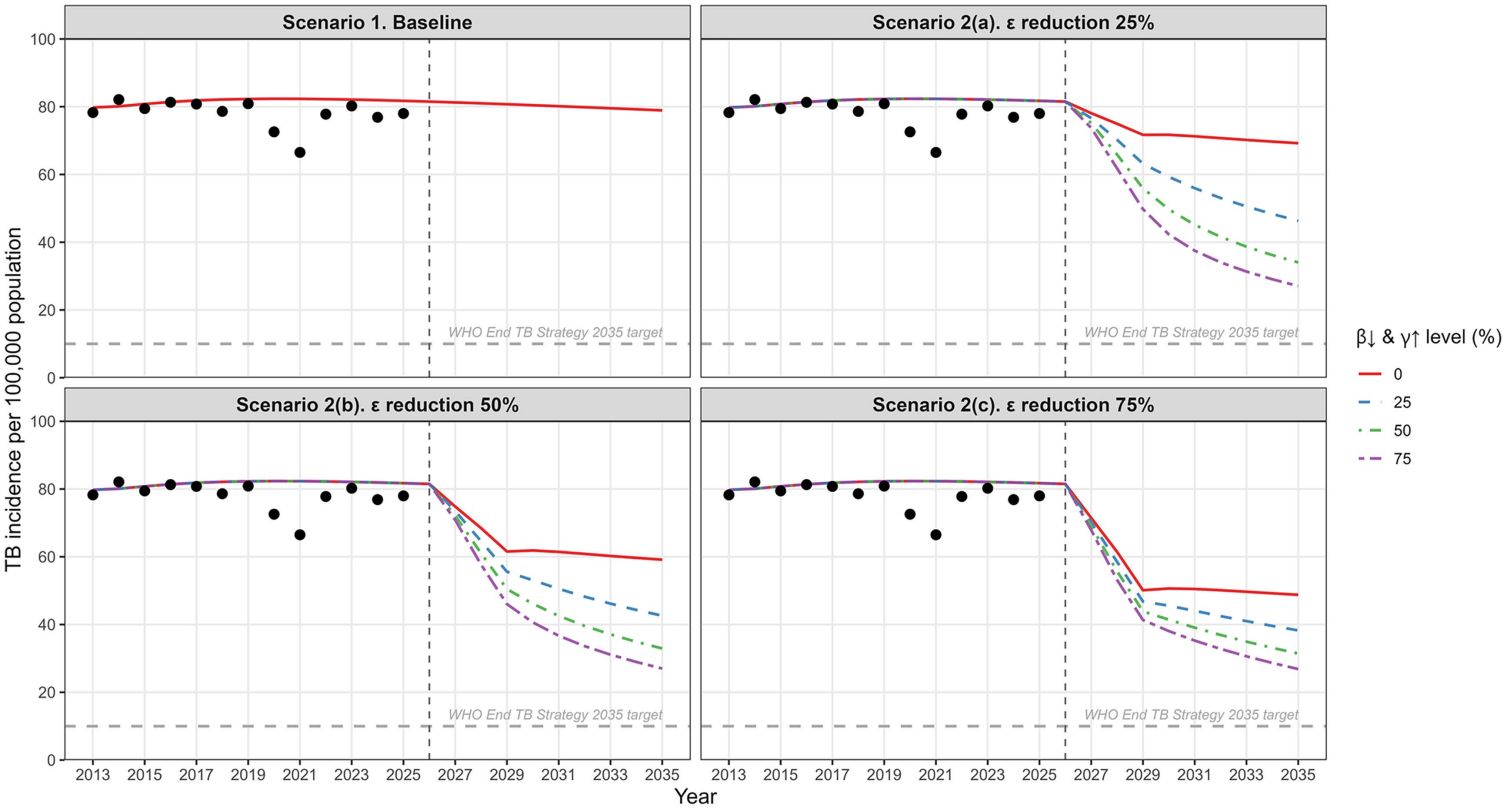
Simulated TB incidence under baseline and intervention scenarios, 2013–2035. Black points represent observed annual TB incidence from 2013 to 2025. Solid and dashed lines show model- simulated TB incidence per 100,000 population using the calibrated SL_F_L_S_IR model. The vertical dashed line indicates the start of intervention scale-up in 2026, with full implementation achieved by the end of 2028. The horizontal dashed line denotes the WHO End TB Strategy 2035 target. Scenario 1 represents the baseline scenario with no parameter changes. Scenario 2(a)–2(c) represent intervention scenarios with fixed reductions in progression from *L_F_* to active TB (*ε* = 25, 50, and 75%, respectively), combined with simultaneous improvements in transmission rate (*β*) and recovery rate (*γ*) at intensities of 0, 25, 50, and 75%.

Figure 5 illustrates the TB incidence trajectory under baseline and intervention simulation scenarios. Under the baseline scenario, TB incidence remained stable over the simulated period with an estimated incidence of 78.91 (95% CI: 70.48–94.54) per 100,000 population in 2035 (Figure 5). Relative to 2015 (reference year for WHO End TB Strategy targets), TB incidence increased slightly by 1.25% in 2030 and decreased slightly by 0.68% in 2035 (Table 4).

**Table 4:** Estimated changes in TB incidence under baseline and intervention scenarios.

Scenario	Reduction in *ε* (%)	Improvements in *β* and *γ* (%)	TB incidence change by 2030 (%)*	TB incidence change by 2035 (%)*
Scenario 1 (Baseline)	0	0	+1.25	−0.68
Scenario 2 (Intervention)				
2(a)	25	0	−9.74	−12.86
		25	−25.37	−41.71
		50	−37.44	−57.18
		75	−46.76	−65.93
2(b)	50	0	−22.14	−25.55
		25	−33.20	−46.37
		50	−41.94	−58.54
		75	−48.86	−65.99
2(c)	75	0	−36.26	−38.60
		25	−42.64	−51.83
		50	−47.84	−60.42
		75	−52.08	−66.21

*Percentage change in TB incidence was calculated relative to the 2015 TB incidence.

#### 3.5.2 Scenario 2 (intervention)

All intervention scenarios led to a reduction in TB incidence, with larger declines observed under higher intervention intensity and over longer time horizons (Figure 5; Table 4). For each fixed level of reduction in progression from latent fast to active TB (*ε*), additional improvements in transmission reduction (*β*) and recovery (*γ*) were associated with progressively larger reductions in TB incidence by 2030 and 2035 (Figure 5; Table 4).

In Scenario 2(a) with a fixed 25% reduction in *ε*, TB incidence reductions by 2035 ranged from 12.86% with no improvement in *β* and *γ* to 65.93% when *β* and *γ* were improved by 75%. In Scenario 2(b) with a fixed 50% reduction in *ε*, TB incidence reductions by 2035 ranged from 25.55% with no improvement in *β* and *γ* to 65.99% when *β* and *γ* were improved by 75%. In Scenario 2(c) with a fixed 75% reduction in *ε*, TB incidence reductions by 2035 ranged from 38.60% with no improvement in *β* and *γ* to 66.21% when *β* and *γ* were improved by 75% (Table 4).

At any given level of *β* and *γ* improvement, increasing the reduction in *ε* from 25 to 75% resulted in additional reductions in TB incidence. When *β* and *γ* were improved by 25%, TB incidence reductions by 2035 were 41.71, 46.37 and 51.83% under *ε* reductions of 25, 50 and 75%, respectively. However, the incremental gains of larger *ε* reductions diminished as *β* and *γ* improvements increased. At the highest *β* and *γ* improvements of 75%, TB incidence reduction by 2035 converged at approximately 65–66% across all *ε* reductions levels of 25, 50 and 75% (Table 4). Although these scenarios produced substantial reductions, none of the simulated scenarios achieved the WHO End TB Strategy target of a 90% reduction in TB incidence by 2035.

## 4 Discussion

This study developed a locally calibrated SL_F_L_S_IR compartmental model using Malaysian TB data 2013–2023 to estimate TB transmission dynamics, with a primary focus on estimating R_0_ and assessing whether simulated TB incidence trajectories under baseline and intervention scenario simulations were compatible with achieving the WHO End TB Strategy targets by 2035. The model demonstrated good alignment with national TB trends over the study period, with explicit adjustment for COVID-19 related disruption to TB detection and reporting. During the COVID-19 pandemic, Malaysia implemented several phases of Movement Control Orders (MCOs) between 2020 and 2022, which restricted population mobility and redirected healthcare resources towards pandemic response. These measures, together with disruptions to routine healthcare services, likely contributed to reduced TB detection and reporting. A study by Oh et al. estimated that TB notifications in Malaysia were under-reported by 12, 21 and 9% in 2020, 2021 and 2022, respectively (31). Although observed reductions in TB notifications during this period may reflect both decreased transmission and under-detection, the relative contribution of these mechanisms remains uncertain (31). To address this, adjusted case counts were incorporated during model calibration to better approximate the underlying TB burden during the pandemic period.

Model calibration and validation showed close correspondence with observed TB trends, with MAPE of 2.49% during model calibration and 3.90% during model validation, suggesting low relative prediction error (42). Other absolute and squared error metrics showed deviations that were small relative to national TB case counts and consistent with overall goodness of fit. The modest increase in error during model validation is in line with the expected increase in uncertainty when estimating beyond the model calibration period. Importantly, observed TB case counts during the validation period remained within the model’s PIs, supporting reasonable short-term consistency of the model for scenario simulations.

The estimated R_0_ of 1.09 indicates that TB transmission in Malaysia remains self-sustaining, with each infectious individual generating slightly one or more secondary case on average. Although direct numerical comparison is limited by differences in model structure and assumptions, the finding of R_0_ slightly above 1 is broadly consistent with Tamhaji and Hamdan who also reported R_0_ of greater than 1 after accounting for the effect of immigration in their model (8). An R_0_ of slightly more than 1.0 is consistent with the observed stability of TB incidence over recent decades and helps explain the limited decline in TB incidence under the no-intervention scenario. This suggests that the existing programme efforts may be sufficient to prevent explosive growth in TB cases but insufficient to drive a substantial and sustained decline in TB incidence. Achieving long-term reductions in TB burden requires interventions that can consistently drive R_0_ below 1. Collectively, these findings suggest that further strengthening of existing TB interventions, together with additional strategies, are required to achieve a substantial reduction in TB incidence.

Scenario simulations from the present study suggested that substantial long-term reductions in TB incidence are most plausible when multiple components of the TB pathway are strengthened concurrently, encompassing both improvements along the TB care cascade and LTBI management. This finding is supported by sensitivity analysis, which showed that simulated TB incidence in 2035 was influenced by *β*, *γ*, *κ*, *L_S_(0)* and *ε.* Across scenarios, reductions in progression from *L_F_* to active TB (*ε*) were associated with declines in TB incidence. However, larger incidence reductions, particularly reductions approaching 50%, were achieved only when reductions in *ε* were combined with improvements in *β* and *γ*. This finding is consistent with prior modelling studies demonstrating that single interventions alone are insufficient to meet End TB Strategy targets (9, 17, 19). Furthermore, greater improvements in transmission and recovery produced larger reductions in simulated TB incidence, underscoring the synergistic effects of integrated programme strategies. Notably, the additional benefit of progressively larger *ε* reductions was not uniform across all scenarios. Reductions in *ε* appeared to provide greater incremental value when improvements in *β* and *γ* were modest, whereas their marginal contribution became smaller when transmission reduction and recovery improvement were already high. This supports a balanced and integrated intervention approach, in which LTBI management is targeted to populations at higher risk of progression and implemented alongside strengthened case detection, early diagnosis, treatment completion, and transmission-reduction strategies.

Evidence from other settings suggests that substantial reductions in TB transmission and incidence are achievable but typically require sustained and coordinated action over many years. Historical declines in England and Wales were associated with both TB-specific measures, including chemotherapy, vaccination, case isolation, and screening, and broader improvements in living conditions, sanitation, nutrition, and public health awareness (43–46). More recent experience from South Korea similarly illustrates that accelerated TB decline is possible when high-coverage interventions, such as public-private engagement, systematic contact investigation, expanded access to TB care, and LTBI management among high-risk groups, are implemented in an integrated and sustained manner (47, 48). These experiences support the interpretation that Malaysia’s progress towards the End TB target will likely require sustained strengthening of TB control together with broader action on population health and social determinants.

In Malaysia, several national strategies are already aligned with these mechanisms. Planned measures by 2030 include strategies targeting transmission reduction and infectious duration, such as systematic TB screening among high-risk populations, deployment of ultra-portable X-ray with artificial intelligence technologies in remote settings, and increased use of WHO-recommended rapid diagnostic tests (41). Complementary strategies aimed at reducing progression from LTBI to active TB include expanded LTBI screening among high-risk groups, such as contacts of bacteriologically confirmed TB cases and individuals with selected comorbidities, along with ensuring strategic accessibility of interferon-gamma release assays and timely initiation of TB preventive treatment (41). Similar integrated approaches also constitute the central roadmaps towards TB elimination in other settings, underscoring the importance of coordinated scale-up of interventions across the TB care pathway, adapted to country-specific context (17, 49).

Although none of the simulation scenarios achieved the WHO End TB target of a 90% reduction in TB incidence by 2035, even under the most intensive intervention assumptions, this finding is consistent with several published studies and should be interpreted in the context of the scenario assumptions (17, 50). In particular, the simulation did not explicitly incorporate interventions targeting reactivation of latent slow TB into active TB, a pathway that is strongly influenced by host-related vulnerabilities and broader population health conditions, rather than TB-specific programme interventions alone. Reactivation risk is closely associated with immunocompromised states, malnutrition, non-communicable diseases (NCDs), behavioural risk factors and socioeconomic disadvantage (51–53). Globally, a substantial proportion of TB incidence is attributable to five major risk factors of undernutrition, diabetes, alcohol use disorders, smoking and HIV (1). If unaddressed, these factors may constrain progress towards the WHO End TB targets. In Malaysia, several of these risk factors have risen over time. The National Health and Morbidity Survey Report 2023 reported that diabetes prevalence among adults increased to 15.6%, compared with 11.2% in 2011 (54). Malaysia also faces a double burden of malnutrition among adolescents, alongside continued challenges related to tobacco use, alcohol consumption and socioeconomic disadvantage, which expose the vulnerable groups to poor living conditions, food insecurity, limited health literacy, poorer access to care and heightened susceptibility to TB infection and progression (54, 55).

Historical and contemporary evidence consistently linked sustained reductions in TB incidence to improvements in socioeconomic conditions, nutrition and overall population health (56–59). As such, progress towards WHO End TB targets is most plausibly achieved through a whole-of-society approach that combines effective TB control with advances in comorbidity management, social protection, housing, education and economic development (50, 53, 60). Within this broader framework, Malaysia has implemented a range of multisectoral strategies that address key upstream determinants of TB risk and reactivation. These include coordinated action on NCDs through the National Strategic Plan for Non-Communicable Diseases (NSP-NCD) 2016–2025, integration of comorbidity management into TB control through risk-based screening guidelines, a comprehensive tobacco control strategy aimed at achieving a tobacco-free nation by 2040, social protection expansion and poverty reduction programmes aimed at improving living conditions and income security (61–63). Collectively, these ongoing multisectoral initiatives provide a supportive foundation for long-term TB burden reduction by addressing both biomedical and social determinant drivers of TB (56).

Several limitations in this study should be acknowledged. The model assumed homogeneous population mixing and does not capture heterogeneity in contact patterns. In addition, active TB is modelled as infectious within a defined period, which may oversimplify variability in infectiousness and care-seeking behaviour. Recovered individuals are also assumed to have complete protection against reinfection within the modelled time frame, which may lead to underestimation of long-term TB incidence. The calibrated values should be interpreted as model-dependent estimates and not direct epidemiological estimates. The intervention scenarios were exploratory counterfactual simulations rather than predictions of guaranteed programme impact. Sustaining the assumed reductions in *β* and *ε* and increases in *γ* until 2035 would require continued programme coverage, financing, and implementation capacity. Future studies should consider incomplete coverage, heterogeneous uptake, and partial waning of intervention effects over time. Despite these limitations, the model provided insights into TB transmission dynamics and the relative impact of key intervention pathways at the population level. Future studies could extend this work by incorporating complementary surveillance metrics, such as the effective reproduction number, to support real-time assessment of transmission intensity and intervention effects.

## 5 Conclusion

This study provides a calibrated SL_F_L_S_IR model with short-term validation showing that TB transmission in Malaysia remains self-sustaining (R_0_ = 1.09). Global sensitivity analysis identified transmission and recovery rate as the main parameters influencing R_0_, while transmission, recovery and latent slow TB progression rates influenced simulated TB incidence. Scenario simulations suggest that continuation of the baseline scenario would produce minimal reduction in TB incidence by 2035, while the most intensive simulated intervention scenario produced a 66.21% reduction relative to 2015, falling short of the WHO End TB target of 90%. These findings indicate that achieving the WHO End TB Strategy target will require integrated action that combines strengthened TB control with improvements in population health and social determinants of TB risk.

## Data Availability

The datasets presented in this article are not readily available due to restrictions applied to the availability of these data. Data were obtained with the permission of the Ministry of Health Malaysia. Requests to access these datasets should be directed to Ministry of Health Malaysia.

## References

[1] World Health Organization. WHO Global Tuberculosis Report 2025. Geneva: World Health Organization (2025).

[2] World Health Organization. *1.1 TB Incidence 2025*. Available online at: https://www.who.int/teams/global-programme-on-tuberculosis-and-lung-health/tb-reports/global-tuberculosis-report-2025/tb-disease-burden/1-1-tb-incidence (Accessed December 17, 2025).

[3] Uplekar M Weil D Lonnroth K Jaramillo E Lienhardt C Dias HM . WHO’s new end TB strategy. Lancet. (2015) 385:1799–801. doi: 10.1016/S0140-6736(15)60570-0, 25814376

[4] Delamater PL Street EJ Leslie TF Yang YT Jacobsen KH. Complexity of the basic reproduction number (R 0). Emerg Infect Dis. (2019) 25:1–4. doi: 10.3201/eid2501.171901, 30560777 PMC6302597

[5] Linka K Peirlinck M Kuhl E. The reproduction number of COVID-19 and its correlation with public health interventions. Comput Mech. (2020) 66:1035–50. doi: 10.1007/s00466-020-01880-8, 32836597 PMC7385940

[6] Van Den Driessche P Watmough J. Reproduction numbers and sub-threshold endemic equilibria for compartmental models of disease transmission. Math Biosci. (2002) 180:29–48. doi: 10.1016/S0025-5564(02)00108-6, 12387915

[7] Ma Y Horsburgh CR White LF Jenkins HE. Quantifying TB transmission: a systematic review of reproduction number and serial interval estimates for tuberculosis. Epidemiol Infect. (2018) 146:1478–94. doi: 10.1017/S0950268818001760, 29970199 PMC6092233

[8] Tamhaji NH Hamdan N‘I. The dynamics of tuberculosis through BSEIR model with immigration in Malaysia. Malays J Fundam Appl Sci. (2023) 19:1176–89. doi: 10.11113/mjfas.v19n6.3063

[9] Kim S De los Reyes VAA Jung E. Country-specific intervention strategies for top three TB burden countries using mathematical model. PLoS One. (2020) 15:e0230964. doi: 10.1371/journal.pone.0230964 32271808 PMC7144981

[10] Martin-Sanchez M Brugueras S De Andrés A Simon P Gorrindo P Ros M . Tuberculosis incidence among infected contacts detected through contact tracing of smear-positive patients. PLoS One. (2019) 14:e0215322. doi: 10.1371/JOURNAL.PONE.0215322, 30986227 PMC6464217

[11] Melsew YA Gambhir M Cheng AC McBryde ES Denholm JT Tay EL . The role of super-spreading events in *Mycobacterium tuberculosis* transmission: evidence from contact tracing. BMC Infect Dis. (2019) 19:3870. doi: 10.1186/S12879-019-3870-1, 30866840 PMC6417041

[12] Borgdorff MW Van Der Werf MJ De Haas PEW Kremer K Van Soolingen D. Tuberculosis elimination in the Netherlands. Emerg Infect Dis. (2005) 11:597–602. doi: 10.3201/EID1104.041103, 15829200 PMC3320334

[13] White PJ Enright MC. Mathematical models in infectious disease epidemiology. Infect Dis. (2012) 1:70. doi: 10.1016/B978-0-323-04579-7.00005-8

[14] Zhao Y Li M Yuan S. Analysis of transmission and control of tuberculosis in mainland China, 2005-2016, based on the age-structure mathematical model. Int J Environ Res Public Health. (2017) 14:14. doi: 10.3390/IJERPH14101192, 28991169 PMC5664693

[15] Zhang J Li Y Zhang X. Mathematical modeling of tuberculosis data of China. J Theor Biol. (2015) 365:159–63. doi: 10.1016/J.JTBI.2014.10.019, 25451959

[16] Liu S Li Y Bi Y Huang Q. Mixed vaccination strategy for the control of tuberculosis: a case study in China. Math Biosci Eng. (2017) 14:695–708. doi: 10.3934/MBE.2017039, 28092959

[17] Houben RMGJ Menzies NA Sumner T Huynh GH Arinaminpathy N Goldhaber-Fiebert JD . Feasibility of achieving the 2025 WHO global tuberculosis targets in South Africa, China, and India: a combined analysis of 11 mathematical models. Lancet Glob Health. (2016) 4:e806–15. doi: 10.1016/S2214-109X(16)30199-1, 27720688 PMC6375908

[18] Chong KC Leung CC Yew WW Zee BCY Tam GCH Wang MH . Mathematical modelling of the impact of treating latent tuberculosis infection in the elderly in a city with intermediate tuberculosis burden. Sci Rep. (2019) 9:4869. doi: 10.1038/s41598-019-41256-4, 30890762 PMC6424958

[19] Dowdy DW Grant AD Dheda K Nardell E Fielding K Moore DAJ. Designing and evaluating interventions to halt the transmission of tuberculosis. J Infect Dis. (2017) 216:S654–61. doi: 10.1093/infdis/jix320, 29112743 PMC5853231

[20] Norlaila MN Hisham H Rostam MI Razab MI Wan Ramli WKH Abdul Manaf ZI. Prediction of tuberculosis disease using SIR model with implementation of Runge-Kutta method in Malaysia. J Math Comput Sci. (2023) 9:109–22. doi: 10.24191/jmcs.v9i2.556

[21] Hamdan NI Latif NF Shaaril N Asmadi NA. Modelling Tuberculosis Transmission with Vaccination in Malaysia: An SVIR Approach. Singapore: Springer Nature Singapore (2025). p. 71–84.

[22] Ministry of Health Malaysia. *Clinical Practice Guidelines. Management of Tuberculosis*. Malaysian Health Technology Assessment Section (MaHTAS) (2021).

[23] Disease Control Division, Ministry of Health Malaysia. *Case Definitions for Infectious Diseases in Malaysia*. Disease Control Division in collaboration with Health Education Division Ministry of Health Malaysia (2017).

[24] Data Catalogue, OpenDOSM. (2025). Available online at: https://open.dosm.gov.my/data-catalogue (Accessed October 22, 2025).

[25] Soetaert K Petzoldt T Setzer RW. Solving differential equations in R: package deSolve. J Stat Softw. (2010) 33:1–25. doi: 10.18637/JSS.V033.I0920808728

[26] Brauer F Castillo-Chavez C Feng Z. Simple compartmental models for disease transmission. Math Models Epidemiol. (2019) 69:21. doi: 10.1007/978-1-4939-9828-9_2

[27] Menzies NA Wolf E Connors D Bellerose M Sbarra AN Cohen T . Progression from latent infection to active disease in dynamic tuberculosis transmission models: a systematic review of the validity of modelling assumptions. Lancet Infect Dis. (2018) 18:e228. doi: 10.1016/S1473-3099(18)30134-8, 29653698 PMC6070419

[28] Price C Nguyen AD. Latent tuberculosis. Rev Chil Enferm Respir. (2024) 28:61–8. doi: 10.4067/s0717-73482012000100009

[29] Das K Murthy BSN Samad SA Biswas MHA. Mathematical transmission analysis of SEIR tuberculosis disease model. Sensors Int. (2021) 2:100120. doi: 10.1016/j.sintl.2021.100120

[30] Tolles J Luong T. Modeling epidemics with compartmental models. JAMA. (2020) 323:2515. doi: 10.1001/jama.2020.8420, 32459319

[31] Oh KH Yanagawa M Morishita F Glaziou P Rahevar K Yadav RP. Changing epidemic of tuberculosis amidst the COVID-19 pandemic in the Western Pacific region: analysis of tuberculosis case notifications and treatment outcomes from 2015 to 2022. Lancet Reg Health West Pac. (2024) 47:101104. doi: 10.1016/J.LANWPC.2024.101104, 38911260 PMC11190483

[32] Marino S Hogue IB Ray CJ Kirschner DE. A methodology for performing global uncertainty and sensitivity analysis in systems biology. J Theor Biol. (2008) 254:178–96. doi: 10.1016/J.JTBI.2008.04.011, 18572196 PMC2570191

[33] Shadabfar M Mahsuli M Sioofy Khoojine A Hosseini VR. Time-variant reliability-based prediction of COVID-19 spread using extended SEIVR model and Monte Carlo sampling. Results Phys. (2021) 26:104364. doi: 10.1016/J.RINP.2021.104364, 34094819 PMC8169594

[34] Akman D Akman O Schaefer E. Parameter estimation in ordinary differential equations modeling via particle swarm optimization. J Appl Math. (2018) 2018:9160793. doi: 10.1155/2018/9160793

[35] Navarro Valencia VA Díaz Y Pascale JM Boni MF Sanchez-Galan JE. Using compartmental models and particle swarm optimization to assess dengue basic reproduction number R0 for the Republic of Panama in the 1999-2022 period. Heliyon. (2023) 9:e15424. doi: 10.1016/j.heliyon.2023.e15424, 37128312 PMC10147988

[36] Musafir RR Anam S. Parameter estimation of COVID-19 compartmental model in Indonesia using particle swarm optimization. J Berkala Epidemiol. (2022) 10:283–92. doi: 10.20473/jbe.V10I32022.283-292

[37] Ozcaglar C Shabbeer A Vandenberg SL Yener B Bennett KP. Epidemiological models of *Mycobacterium tuberculosis* complex infections. Math Biosci. (2012) 236:77–96. doi: 10.1016/j.mbs.2012.02.003, 22387570 PMC3330831

[38] Chowell G Bleichrodt A Luo R. Parameter estimation and forecasting with quantified uncertainty for ordinary differential equation models using QuantDiffForecast: a MATLAB toolbox and tutorial. Stat Med. (2024) 43:1826–48. doi: 10.1002/sim.10036, 38378161 PMC11031352

[39] Chowell G. Fitting dynamic models to epidemic outbreaks with quantified uncertainty: a primer for parameter uncertainty, identifiability, and forecasts. Infect Dis Model. (2017) 2:379–98. doi: 10.1016/j.idm.2017.08.001, 29250607 PMC5726591

[40] Blower SM Dowlatabadi H. Sensitivity and uncertainty analysis of complex models of disease transmission: an HIV model, as an example. Int Stat Rev. (1994) 62:229. doi: 10.2307/1403510

[41] Disease Control Division, Ministry of Health Malaysia. *National Strategic Plan to End TB (2021–2030)* (2021).

[42] Klimberg RK Sillup GP Boyle KJ Tavva V. Forecasting Performance Measures – what are their Practical Meaning? Leeds: Emerald Group Publishing Limited (2010). p. 137–47.

[43] Vynnycky E Fine PEM. The long-term dynamics of tuberculosis and other diseases with long serial intervals: implications of and for changing reproduction numbers. Epidemiol Infect. (1998) 121:309–24. doi: 10.1017/S0950268898001113, 9825782 PMC2809528

[44] Holloway-Kew KL Henneberg M. Dynamics of tuberculosis infection in various populations during the 19th and 20th century: the impact of conservative and pharmaceutical treatments. Tuberculosis. (2023) 143:102389. doi: 10.1016/J.TUBE.2023.102389, 38012934

[45] Glaziou P Floyd K Raviglione M. Trends in tuberculosis in the UK. Thorax. (2018) 73:702–3. doi: 10.1136/THORAXJNL-2018-211537, 29674388 PMC6204963

[46] Carwile ME Hochberg NS Sinha P. Undernutrition is feeding the tuberculosis pandemic: a perspective. J Clin Tuberc Other Mycobact Dis. (2022) 27:100311. doi: 10.1016/J.JCTUBE.2022.100311, 35313724 PMC8928739

[47] Cho KS. Tuberculosis control in the Republic of Korea. Epidemiol Health. (2018) 40:e2018036. doi: 10.4178/epih.e2018036, 30081621 PMC6335497

[48] Min J Jeong Y Kim HW Kim JS. Tuberculosis notification and incidence: Republic of Korea, 2023. Tuberc Respir Dis (Seoul). (2025) 88:606–9. doi: 10.4046/trd.2025.0022, 40384061 PMC12235280

[49] Feng Q Zhang G Chen L Wu H Yang Y Gao Q . Roadmap for ending TB in China by 2035: the challenges and strategies. Biosci Trends. (2035) 18:11–20. doi: 10.5582/bst.2023.01325, 38325824

[50] Ayenew B Belay DM Gashaw Y Gimja W Gardie Y. WHO’S end of TB targets: unachievable by 2035 without addressing under nutrition, forced displacement, and homelessness: trend analysis from 2015 to 2022. BMC Public Health. (2024) 24:961. doi: 10.1186/s12889-024-18400-5, 38575958 PMC10996214

[51] Duarte R Lönnroth K Carvalho C Lima F Carvalho ACC Muñoz-Torrico M . Tuberculosis, social determinants and co-morbidities (including HIV). Pulmonology. (2018) 24:115–9. doi: 10.1016/j.rppnen.2017.11.003, 29275968

[52] Ai J-W Ruan Q-L Liu Q-H Zhang W-H. Updates on the risk factors for latent tuberculosis reactivation and their managements. Emerg Microbes Infect. (2016) 5:1–8. doi: 10.1038/emi.2016.10, 26839146 PMC4777925

[53] Chakaya JM Harries AD Marks GB. Ending tuberculosis by 2030—pipe dream or reality? Int J Infect Dis. (2030) 92:S51–4. doi: 10.1016/j.ijid.2020.02.021, 32114202

[54] Institute for Public Health (IKU) *National Health and Morbidity Survey 2023: Non-Communicable Diseases and Healthcare Demand: Technical Report* (2024).

[55] Department of Statistics Malaysia. *Statistics on Poverty. Poverty in Malaysia, 2024*. (2025). Available online at: https://www.dosm.gov.my/portal-main/release-content/poverty-in-malaysia-2024 (Accessed December 24, 2025).

[56] Lönnroth K Castro KG Chakaya JM Chauhan LS Floyd K Glaziou P . Tuberculosis control and elimination 2010–50: cure, care, and social development. Lancet. (2010) 375:1814–29. doi: 10.1016/S0140-6736(10)60483-7, 20488524

[57] Franco JV Bongaerts B Metzendorf M-I Risso A Guo Y Peña Silva L . Undernutrition as a risk factor for tuberculosis disease. Cochrane Database Syst Rev. (2024) 6:CD015890. doi: 10.1002/14651858.CD015890.pub2, 38860538 PMC11165671

[58] Hayashi S Chandramohan D. Risk of active tuberculosis among people with diabetes mellitus: systematic review and meta-analysis. Trop Med Int Health. (2018) 23:1058–70. doi: 10.1111/TMI.13133 30062731

[59] Imtiaz S Shield KD Roerecke M Samokhvalov AV Lönnroth K Rehm J. Alcohol consumption as a risk factor for tuberculosis: meta-analyses and burden of disease. Eur Respir J. (2017) 50:1700216. doi: 10.1183/13993003.00216-2017, 28705945 PMC5540679

[60] John CA. Realizing the World Health Organization’s end TB strategy (2016–2035): how can social approaches to tuberculosis elimination contribute to Progress in Asia and the Pacific? Trop Med Infect Dis. (2019) 4:28. doi: 10.3390/tropicalmed4010028, 30764510 PMC6473717

[61] Ministry of Health Malaysia. *Prevention and Control of Noncommunicable Diseases in Malaysia: The Case for Investment* (2024).

[62] Public Health Department, Ministry of Health Malaysia. *The National Strategic Plan For Non-Communicable Disease (NSP-NCD) 2016–2025* (2016)

[63] Ministry of Health Malaysia. *The National Strategic Plan for the Control of Tobacco and Smoking Products 2021–2030* (2021).

