# Supplementary material for: Modelling tuberculosis transmission dynamics in Malaysia
Source: Front Public Health. 2026 Jul 14;14:1843471. doi: 10.3389/fpubh.2026.1843471 (PMC13407623; doi:10.3389/fpubh.2026.1843471)
Supplement: Supplementary file 1 [file Data_Sheet_1.PDF]

Supplementary Table S1. Parameter ranges and candidate parameter values in Monte Carlo exploration

Parameter	Description	Sampling distribution (range)	Converted rate range	Source	Candidate parameter value
ε	Rate of progression from latent fast to active TB	Uniform (0.25,2) years	0.50-4.00 $year^{-1}$	[1–3]	0.65
κ	Rate of progression from latent slow to active TB	Uniform (2,75) years	0.013-0.50 $year^{-1}$	[3–5]	0.014
β	Transmission rate	Derived from sampled R_0 where R_0 ~ Uniform (0.5, 2.0)	Derived	Estimated	1.16
γ	Rate of progression from active TB to recovery	Uniform (0.083,3) years	0.33–12.00 $year^{-1}$	[6–8]	0.76
$N(0)$	Initial total population size in year 2013	30,200,000 individuals	-	[5]	30,200,000
$L_F(0)$	Initial value of latent fast compartment	Uniform (30,000, 200,000) individuals	-	Estimated	33,501
$L_S(0)$	Initial value of latent slow compartment	Uniform (100,000, 7,550,000) individuals	-	Estimated	517,981
$I(0)$	Initial value of infectious TB compartment	Uniform (10,000, 50,000) individuals	-	Estimated	22,592
$R(0)$	Initial value of recovered compartment	200,000 individuals	-	Assumed	200,000
$S(0)$	Initial value of susceptible compartment	Derived as S(0)=N(0)-LF(0)-LS(0)-I(0)-R(0)	-	Derived	29,425,926

References

[1] Menzies NA, Wolf E, Connors D, Bellerose M, Sbarra AN, Cohen T, et al. Progression from latent infection to active disease in dynamic tuberculosis transmission models: a systematic review of the validity of modelling assumptions. Lancet Infect Dis 2018;18:e228. https://doi.org/10.1016/S1473-3099(18)30134-8.

[2] Behr MA, Edelstein PH, Ramakrishnan L. Revisiting the timetable of tuberculosis. BMJ 2018:k2738. https://doi.org/10.1136/bmj.k2738.

[3] Price C, Nguyen AD. Latent Tuberculosis. Revista Chilena de Enfermedades Respiratorias 2024;28:61–8. https://doi.org/10.4067/s0717-73482012000100009.

[4] Kiazyk S, Ball T. Latent tuberculosis infection: An overview. Canada Communicable Disease Report 2017;43:62–6. https://doi.org/10.14745/ccdr.v43i34a01.

[5] Data Catalogue | OpenDOSM n.d. https://open.dosm.gov.my/data-catalogue (accessed October 22, 2025).

[6] American Thoracic Society, Centers for Disease Control and Prevention, Infectious Diseases Society of America. Controlling Tuberculosis in the United States. Am J Respir Crit Care Med 2005;172:1169–227. https://doi.org/10.1164/rccm.2508001.

[7] Tiemersma EW, van der Werf MJ, Borgdorff MW, Williams BG, Nagelkerke NJD. Natural History of Tuberculosis: Duration and Fatality of Untreated Pulmonary Tuberculosis in HIV Negative Patients: A Systematic Review. PLoS One 2011;6:e17601. https://doi.org/10.1371/journal.pone.0017601.

[8] Goswami N, Reed C. Duration of Effective Tuberculosis Treatment, Not Acid-Fast Bacilli (AFB) Smear Status, as the Determinant for De-isolation in Community Settings. Clinical Infectious Diseases 2024. https://doi.org/10.1093/cid/ciae198.

Supplementary Method S1. Derivation of the basic reproduction number using the next-generation matrix approach

The basic reproduction number, R_0, for the SL$_F$L$_S$IR model was derived using the next-generation matrix approach.

The infected-state vector was defined as:

$$x = (L_F, L_S, I)^T$$

where L_F represents latent fast TB infection, L_S represents latent slow TB infection, and I represents active infectious TB. Although individuals in L_F and L_S are not infectious, these compartments were included in the infected-state subsystem because they represent infected individuals who may subsequently progress to active TB.

The relevant infected-state equations are:

$$\frac{dL_F}{dt} = \beta I \frac{S}{N} - (\varepsilon + \nu + \mu)L_F$$

$$\frac{dL_S}{dt} = \nu L_F - (\kappa + \mu)L_S$$

$$\frac{dI}{dt} = \varepsilon L_F + \kappa L_S - (\gamma + \mu + m)I$$

At the disease-free equilibrium, $L_F^* = L_S^* = I^* = 0$ and $S^*/N = 1$.. The new infection vector, $F(x)$, is:

$$F(x) = \begin{pmatrix} \beta I \frac{S}{N} \\ 0 \\ 0 \end{pmatrix}$$

The transition vector, $V(x)$, representing transfers between infected compartments and removals from infected states, is:

$$V(x) = \begin{pmatrix} (\varepsilon + \nu + \mu)L_F \\ (\kappa + \mu)L_S - \nu L_F \\ (\gamma + \mu + m)I - \varepsilon L_F - \kappa L_S \end{pmatrix}$$

The Jacobian matrices of $F(x)$and $V(x)$, evaluated at the disease-free equilibrium, are:

$$F = \begin{pmatrix} 0 & 0 & \beta \\ 0 & 0 & 0 \\ 0 & 0 & 0 \end{pmatrix}$$

and

$$V = \begin{pmatrix} \varepsilon + \nu + \mu & 0 & 0 \\ -\nu & \kappa + \mu & 0 \\ -\varepsilon & -\kappa & \gamma + \mu + m \end{pmatrix}$$

Let:

$a = \varepsilon + \nu + \mu, b = \kappa + \mu, c = \gamma + \mu + m$

Then:

$$V = \begin{pmatrix} a & 0 & 0 \\ -\nu & b & 0 \\ -\varepsilon & -\kappa & c \end{pmatrix}$$

and its inverse is:

$$V^{-1} = \begin{pmatrix} \frac{1}{a} & 0 & 0 \\ \frac{\nu}{ab} & \frac{1}{b} & 0 \\ \frac{\varepsilon b + \kappa\nu}{abc} & \frac{\kappa}{bc} & \frac{1}{c} \end{pmatrix}$$

Thus, the next-generation matrix is:

$$FV^{-1} = \begin{pmatrix} \frac{\beta(\varepsilon b + \kappa\nu)}{abc} & \frac{\beta\kappa}{bc} & \frac{\beta}{c} \\ 0 & 0 & 0 \\ 0 & 0 & 0 \end{pmatrix}$$

The basic reproduction number is the spectral radius of the next-generation matrix:

$$R_0 = \rho(FV^{-1})$$

Since FV^{-1} has only one non-zero eigenvalue:

$$R_0 = \frac{\beta(\varepsilon b + \kappa\nu)}{abc}$$

Substituting $a = \varepsilon + \nu + \mu$, $b = \kappa + \mu$, and $c = \gamma + \mu + m$:

$$R_0 = \frac{\beta[\varepsilon(\kappa + \mu) + \kappa\nu]}{(\varepsilon + \nu + \mu)(\kappa + \mu)(\gamma + \mu + m)}$$

Equivalently:

$$R_0 = \frac{\beta}{\gamma + \mu + m} \cdot \left[\frac{\varepsilon}{\varepsilon + \nu + \mu} + \frac{\nu}{\varepsilon + \nu + \mu} \cdot \frac{\kappa}{\kappa + \mu}\right]$$

In this expression, $\beta/(\gamma + \mu + m)$ represents the expected number of new infections generated during the infectious period, while the bracketed term represents the probability that a newly infected individual progresses to active TB through either the latent fast pathway or the latent slow pathway.
